# Role of MRI in Differentiating Various Posterior Cranial Fossa Space-Occupying Lesions Using Sensitivity and Specificity: A Prospective Study

**DOI:** 10.7759/cureus.16336

**Published:** 2021-07-12

**Authors:** Prabhakaran Tamilchelvan, Deb K Boruah, Bidyut B Gogoi, Rudrakanta Gogoi

**Affiliations:** 1 Radiodiagnosis, Assam Medical College and Hospital, Dibrugarh, IND; 2 Radiodiagnosis, Tezpur Medical College, Tezpur, IND; 3 Pathology, Assam Medical College and Hospital, Dibrugarh, IND

**Keywords:** magnetic resonance imaging (mri), diffusion-weighted imaging (dwi), magnetic resonance spectroscopy (mrs), tumor, posterior fossa

## Abstract

Background: Any abnormal space-occupying posterior fossa lesion may directly involve the vital structures like the brain stem, cranial nerves, cerebellum, vertebrobasilar artery, and venous sinuses, which makes the surgical approach and total excision very difficult. Hence for these reasons, precise evaluation of posterior fossa lesion with MRI is a must to visualize the vital structures, which helps in planning and safe surgery.

Objective: This study aimed to evaluate the added value of diffusion-weighted imaging and magnetic resonance spectroscopy in the localization, extension, characterization, differentiation of various posterior fossa space-occupying lesions, and correlating with the histopathological result.

Materials and methods: This prospective study comprised of 40 patients who were suspected with posterior fossa space-occupying lesions on basis of clinical features or on CT scan. All patients were evaluated using conventional as well as newer MRI techniques using Siemens 1.5 Tesla MRI scanner (Siemens Medical System, Erlangen, Germany). Diffusion-weighted imaging (DWI) was done in all patients and magnetic resonance spectroscopy (MRS) was done in 27 patients. Based on the MRI findings, various posterior fossa lesions were classified as neoplastic or non-neoplastic. The neoplastic lesions were further classified as benign and malignant. The MRI findings were correlated with histopathological findings or follow-up.

Statistical analysis: Independent sample t-test was used to compare the mean apparent diffusion coefficient (ADC) values of various posterior fossa space-occupying lesions. Receiver operating characteristic (ROC) curve analysis was done to determine the optimal cut-off mean ADC values and choline/creatinine (Cho/cr) ratios for various benign and malignant posterior fossa tumors.

Results: Of 40 patients with posterior fossa lesions, 23 were males and 17 were females with a mean age of 34.67±1.93[SD] years. Metastases were the most common posterior fossa lesions in our study sample and found in seven patients (17.5%) followed by vestibular schwannomas and brainstem gliomas in five patients (12.5%) each, demyelinating lesion in four patients (10%), tubercular abscess in three patients (7.5%), hemangioblastoma, tuberculoma, arachnoid cyst, epidermoid cyst, pilocytic astrocytoma, low-grade glioma in two patients (5%) each, meningioma, medulloblastoma, pyogenic abscess and high-grade glioma in one patient (2.5%) each. The mean ADC value of benign tumors was higher than that of malignant tumors and this difference was found to be significant (p = 0.019). The cut-off ADC value 1.022 x 10^-3^mm^2^/s had a sensitivity of 78.6% and specificity of 66.7%. MRS played important role in differentiating neoplastic from non-neoplastic lesions and benign from malignant tumors. The cut-off Cho/cr ratio of 1.25 had a sensitivity of 66.7%, specificity of 85.7% to differentiate benign from malignant tumors.

Conclusion: Conventional MRI sequences able to diagnose most of the benign-appearing lesions of posterior fossa, however, adding advanced MRI sequences like diffusion-weighted imaging and MR spectroscopy helps us to differentiate and diagnose various posterior fossa lesions even closer to the actual histopathological diagnosis.

## Introduction

The posterior fossa of the brain consists of vital structures like the brainstem, cerebellum, lower cranial nerves, vertebrobasilar arteries, and venous sinuses. The posterior fossa is the spaces above the foramen of the magnum and below the tentorial cerebelli [1]. Computed tomography of the posterior fossa is limited by the beam hardening artifacts and which adversely hamper the quality of images. MRI has played an increasingly important role and is the investigation of choice in patients suspected of harboring lesions in the posterior fossa. T1-weighted images provide excellent anatomical details while T2-weighted images provide pathological information [2].

Conventional MR imaging provides diagnosis with a success rate of 30-90%. However, advanced MRI sequences like diffusion-weighted imaging (DWI), MR perfusion, and magnetic resonance spectroscopy (MRS) can provide immense value in the characterization and diagnosis of various posterior fossa space-occupying lesions [3,4].

DWI is helpful in distinguishing between the brain abscesses, necrotic, and cystic neoplasms on MRI. Increased tumor cellularity in neoplasm causes a relative reduction in the apparent diffusion coefficient (ADC) values and that helps in differentiation between the medulloblastoma, ependymoma, and pilocytic astrocytoma [3]. By adding DWI and MR spectroscopy over conventional MRI can negate the need for biopsy in various posterior fossa tumors [3]. DWI is an excellent tool used in differentiating epidermoid from arachnoid cysts even difficult to differentiate with conventional MR sequences [4].

MRS gives information related to cell membrane proliferation, neuronal damage, energy metabolism, and necrotic transformation of brain or tumor tissues. MRS gives information at the molecular level rather than structural and anatomic imaging of tissues and organs by identifying various chemical metabolites [5,6]. MR spectroscopy can differentiate between the non-neoplastic and neoplastic lesions, benign and malignant brain lesions [6,7].

This study aimed to evaluate the added value of diffusion-weighted imaging and MRS in the localization, extension, characterization, differentiation of various posterior fossa space-occupying lesions, and correlating with the histopathological result.

## Materials and methods

Study design

**Figure 1:**
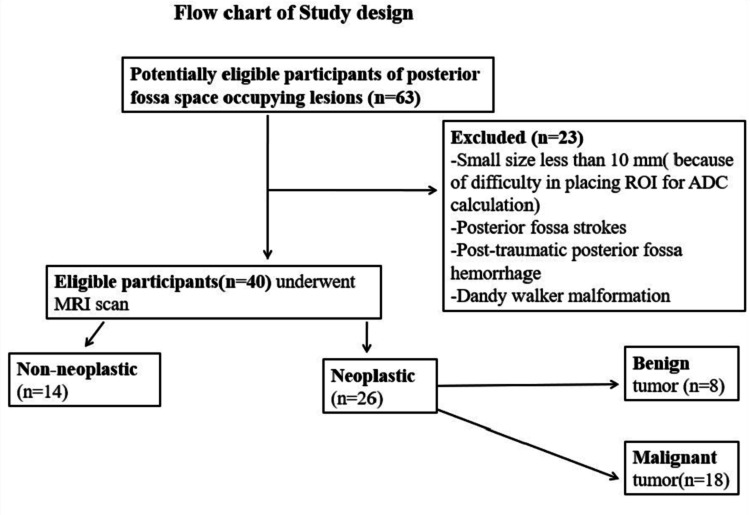
Flow chart of the study design. ROI: region of interest; ADC: apparent diffusion coefficient

After approval from the institutional ethics review committee, a hospital-based cross-sectional prospective study was conducted. This study group comprised of 40 patients with CT proven or clinical suspicion of posterior fossa space-occupying lesion presented to the Department of Radiodiagnosis in a tertiary care center of Northeast India from July 2017 to June 2018. Informed consent was obtained from the patient/parents/guardian before undergoing an MRI scan (Figure 1). Patients with posterior fossa stroke, patients with a history of head trauma, patients with congenital posterior fossa malformation like Dandy-Walker cyst, inadequate or noisy MR spectroscopy, and lesion size less than 10 mm were excluded from the study.

Test method

All patients were subjected to an MRI scan of the brain in a supine position in Siemens Magnetom Avanto 1.5 Tesla MR scanner (Siemens Medical System, Erlangen, Germany). Conventional MR sequences like axial T1WI, T2WI, fluid-attenuated inversion recovery (FLAIR), susceptibility-weighted image (SWI), diffusion-weighted image (DWI), sagittal T1W1, and coronal T2WI sequences were obtained using 4-5 mm slice thickness. DWI using echo-planar imaging (EPI) was done in all the patients before contrast administration. Post gadolinium fat-suppressed T1W images were obtained in different planes. The MR spectroscopy data were obtained in 27 patients only. Both single and multi-voxel techniques (chemical shift imaging {CSI} sequence) were employed in our study with TE 135 msec and 30 msec. Both stimulated echo acquisition mode (STEAM) and point resolved spectroscopy (PRESS) techniques were employed. Various MRI sequences are shown in (Table 1).

**Table 1:** MR imaging sequences of the brain in posterior fossa lesions. FS: fat-suppressed; TE: time of echo; TR: time of relaxation; FLAIR: fluid-attenuated inversion recovery; DWI: diffusion-weighted image; SWI: susceptibility-weighted image

Parameters	T2W	FLAIR	T1W	Coronal T2W	Sagittal T1W	DWI	SWI	Post-gadolinium fat-suppressed T1W
Imaging plane	axial	axial	axial	coronal	Sagittal	axial	axial	axial, coronal & sagittal
TE/TR (msec)	100/3500	90/3500 (TI=2500)	7-9/500	90/3600	90/3600, TI=160	90/3900	200/1500	10/550
FOV (cm)	190-200	190-200	190-240	200-240	190-200	190-200	190-200	190-200
Slice thickness (mm)	4	4	5	4	4	4	3	3.5
Interslice gap (mm)	0.5	0.5	1.5	1	0.3	0.4	1.5	0.3
No of slices	30	30	21	25	25	30	120	30
Matrix	272 × 215	212 × 186	234 × 384	234 × 384	252 × 216	80 × 79	220 × 220	256 × 256
Flip angle	90^0^	90^0^	90^0^	90^0^	90^0^	90^0^	90^0^	90^0^
Echo train length	19	20	13	20	22	-	94	4
b-value (s/mm^2^)	-	-		-	-	1000	-	-

Analysis

Two radiologists, one having experience of neuroradiology for 13 years and another having four years of experience prospectively analyzed the MRI images. The two radiologists were blinded to the clinical history or previous radiological reports of the patients. The size of the posterior fossa space-occupying lesion and ADC values were calculated independently by the two radiologists and mean ADC values were used for the results. The MR spectroscopy parameters especially the choline, N-acetyl aspartate (NAA), lipid, lactate, aminoacid peaks, and choline/creatinine ratio were analyzed independently. 

Analysis of Conventional MR Images

We evaluated the following characteristics of a space-occupying lesion in the posterior fossa like lesion sizes, locations, margins, T1WI and T2WI appearances, perilesional edema, and lesion heterogeneity. The posterior fossa lesion size was obtained from the largest dimension of a lesion. The location of a posterior fossa lesion is identified according to the involvement of posterior fossa structures. Margins of space-occupying lesion in posterior fossa classified into well-defined and ill-defined. The “well-defined” margin was considered when the margin of space-occupying lesion was differentiated from surrounding structures. An “ill-defined” lesion was considered when the margin of a lesion was ill-defined. Lesion heterogeneity was considered when there were mixed-signal intensities on T1WI or T2W images. 

ADC Calculation Analysis

Minimum, maximum, and mean ADC values of a posterior fossa space-occupying lesion were calculated from placing either round or elliptical region of interests (ROIs) over the solid, enhancing, non-necrotic, and/or DWI restricted part of a solid enhancing lesion. In a peripherally enhancing cystic lesion, ROIs were placed in the enhancing wall of the lesions. In the case of cystic lesions and enhancing mural nodules, ROIs were placed in the enhancing mural nodule. ADC values were measured in the operating system console using multiple uniform sizes (area: minimum 10 mm^2^, maximum 50 mm^2^). We divided the lesion into four quadrants and calculated the ADC values from four quadrants and the final mean ADC value was calculated. In patients with multiple posterior fossa lesions, the largest lesion was selected for calculation of the mean ADC value.

MR Spectroscopy Data Analysis

Appropriate-sized voxels were chosen to analyze the metabolites with MR spectroscopy. MR spectroscopy spectra were analyzed for various metabolites like choline, creatinine, N-acetyl aspartate, lactate, lipid, alanine, and amino acids. Metabolite ratios like Cho/cr were generated. Mean values of the Cho/cr ratio of different lesions were calculated and compared with a final diagnosis. Mean Cho/cr ratio of neoplastic and non-neoplastic lesions, benign and malignant tumors were then compared and their statistical significance was calculated.

Histopathological Analysis/Final Diagnosis

The final diagnosis was achieved by typical MR imaging findings in n= 15 patients, histopathological diagnosis of posterior fossa space-occupying lesion was established on surgically resected specimens in n=19 patients, and follow-up post-treatment scan in n= 6 patients.

Statistical Analysis

All the statistical analysis was performed using Statistical Package for Social Sciences (SPSS) version 16 (Armonk, NY: IBM Corp.). The mean ADC values of various lesions were calculated by an independent sample t-test. The optimal cut-off mean ADC values and Cho/cr ratio for various posterior fossa tumors were obtained from receiver operating characteristic (ROC) curve analysis.

## Results

Of 40 patients with posterior fossa lesions, 23 were males and 17 were females with a mean age of 34.67±1.93[SD] years. Maximum nine patients (22.5%) each were in the age group of 11-20 years and 31-40 years followed by seven patients (17.5%) in the age group of 41-50 years and five patients (12.5%) in the age group of 21-30 years. Only seven patients (17.5%) were above the age of 50 years. Headache and vomiting were the most common presenting symptoms found in 24 patients (60%).

Thirty patients (75%) had intra-axial space-occupying lesions while 10 patients (25%) had extra-axial lesions. In the intra-axial compartment, the cerebellum is the most commonly involved in 19 patients (47.5%) followed by brain stem in six (15%) patients. In the extra-axial compartment, cerebello-pontine (CP) angle cistern was most commonly involved in five patients (12.5%).

Metastases were the most common posterior fossa lesions in our study sample and found in seven patients (17.5%) followed by vestibular schwannomas and brainstem gliomas in five patients (12.5%) each, demyelinating lesion in four patients (10%), tubercular abscess in three patients (7.5%), hemangioblastoma, tuberculoma, arachnoid cyst, epidermoid cyst, pilocytic astrocytoma, low-grade glioma in two patients (5%) each, meningioma, medulloblastoma, pyogenic abscess and high-grade glioma in one patient (2.5%) each. The different posterior fossa lesions with their mean ADC values are shown in Table 2.

**Table 2:** Various posterior fossa space-occupying lesions with their mean ADC values in 40 patients. ADC: apparent diffusion coefficient

Posterior fossa lesion	Number/percentage of cases	Mean ADC value × 10^-3 ^mm^2^/s
Metastases	7(17.5%)	0.886 ± 0.21
Brainstem glioma	5(12.5%)	1.093 ± 0.36
Vestibular schwannoma	5(12.5%)	1.121 ± 0.41
Demyelinating lesions	4(10%)	0.893 ± 0.02
Tubercular abscess	3(7.5%)	0.697 ± 0.14
Hemangioblastoma	2 (5%)	1.604 ± 0.24
Tuberculoma	2(5%)	1.019 ± 0.08
Arachnoid cyst	2(5%)	3.095 ± 0.10
Epidermoid cyst	2(5%)	0.714 ± 0.11
Pilocytic astrocytoma	2(5%)	1.615 ± 0.03
Low-grade glioma	2(5%)	1.232 ± 0.27
Meningioma	1(2.5%)	0.843
Medulloblastoma	1(2.5%)	0.680
Pyogenic abscess	1(2.5%)	0.384
High-grade cerebellar glioma	1(2.5%)	0.708
Benign tumor	8(20%)	1.279±0.38
Malignant tumor	18(45%))	0.954 ±0.27

**Figure 2:**
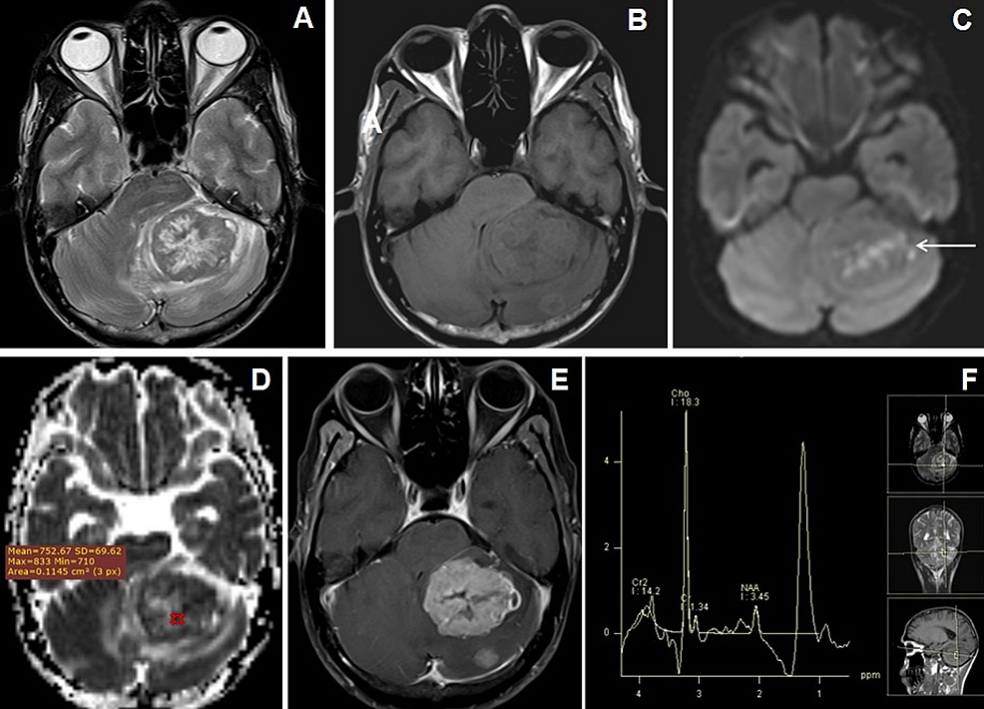
Cranial MRI of a 49 years male who presented with seizures and headache with cerebellar metastasis from adenocarcinoma of lung. (A) Axial T2WI image shows central T2W hyperintense and peripheral isointense larger lesion in the left cerebellar hemisphere with perifocal edema. (B) Axial T1WI image shows isointense signal intensities within the larger lesion. Another smaller isointense nodular lesion was seen in the left cerebellar hemisphere posterior to the larger one. (C and D) Axial DWI and ADC map images show patchy diffusion restriction (←arrow) with low to variable ADC values. (E) Axial T1W post-contrast image shows heterogeneous enhancement of the larger lesion with central necrosis along with another solid enhancing nodule in the left cerebellum posterior to the larger one. (F) MR spectroscopy images at TE-135 ms show increased choline and lipid-lactate peaks with reduction of NAA peak. NAA: N-acetyl aspartate; DWI: diffusion-weighted imaging; ADC: apparent diffusion coefficient

**Figure 3:**
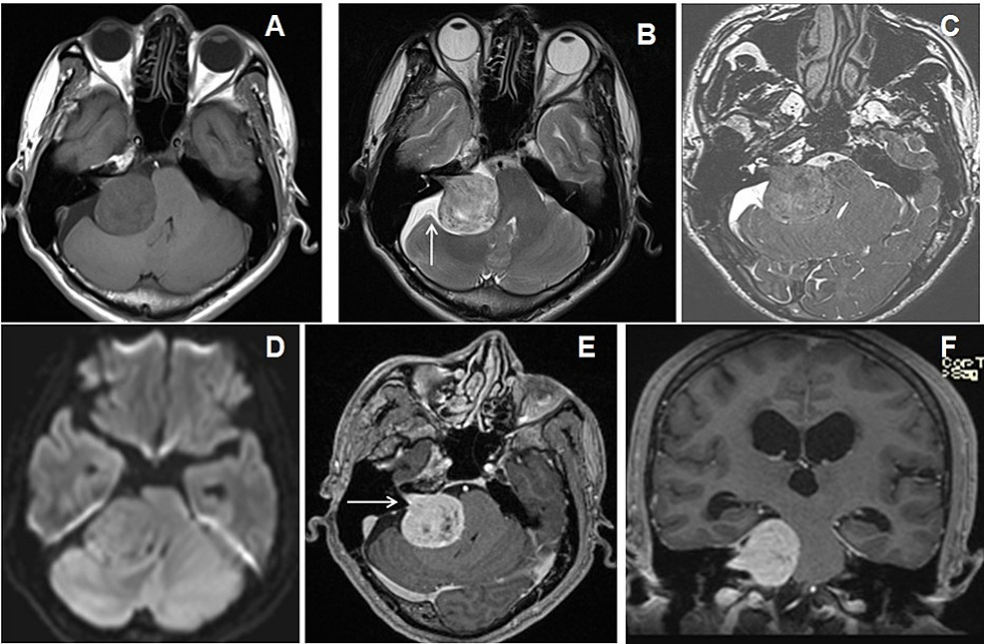
Cranial MRI of a 50-year-old male patient with vertigo and ataxia due to right-sided vestibular schwannoma. (A and B) Axial T1WI and T2WI images show an extra-axial T1W isointense and T2 mixed signal intensity extra-axial lesion in right C-P angle cistern with CSF cleft (↑arrow). (C) 3D-CISS image shows the tumoral extension into the right IAC. (D) DWI image does not show diffusion restriction. (E and F) Axial and coronal T1W post-contrast images show moderate homogenous enhancement of the lesion (→arrow) with fine non-enhancing cystic spaces. CSF: cerebrospinal fluid; 3D-CISS: three-dimensional constructive interference in steady state; DWI: diffusion-weighted image

Posterior fossa metastasis was the most common intra-axial lesion in posterior fossa lesion (Figures 2A-2F) while vestibular schwannoma was the most common extra-axial tumor (Figures 3A-3F).

**Figure 4:**
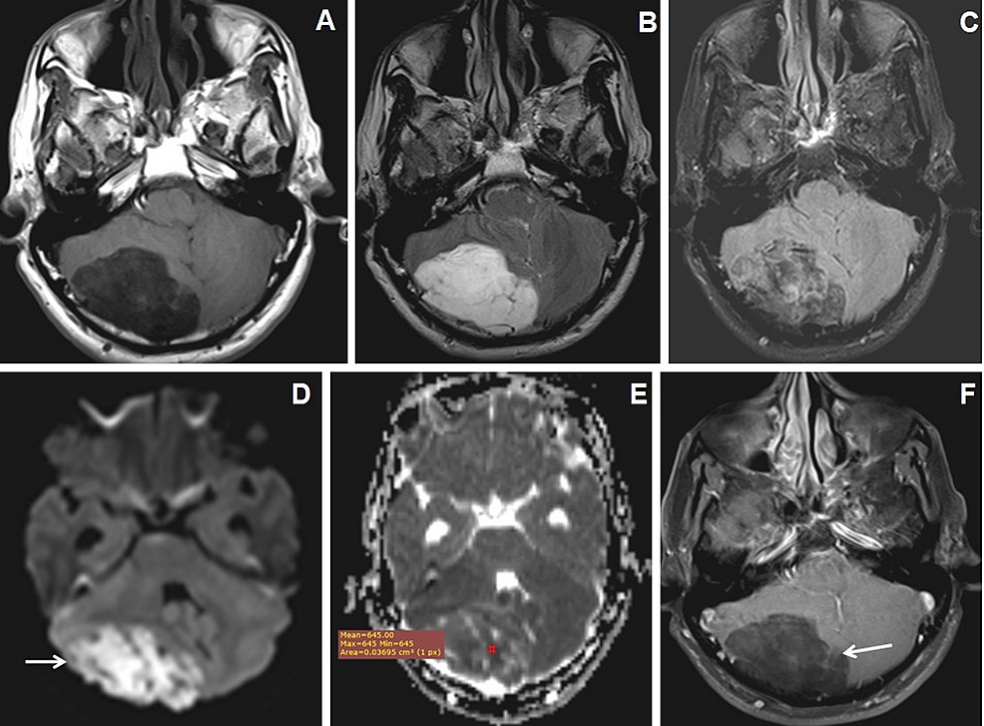
Cranial MRI of a 41-year-old male patient with headache due to epidermoid cyst. (A and B) Axial T1WI and T2WI images show an extra-axial heterogeneous appearing T1WI hypointense and T2WI hyperintense lesion over the right cerebellar convexity with intervening T2W hypointense septae. (C) FLAIR image shows incomplete suppression of the cyst. (D and E) Axial DWI and ADC map images show restricted diffusion (→arrow) with variable ADC values. (F) Axial T1W post-contrast image shows no enhancement (←arrow). FLAIR: fluid-attenuated inversion recovery; DWI: diffusion-weighted imaging; ADC: apparent diffusion coefficient

DWI with ADC values clearly differentiating arachnoid cyst from the epidermoid cyst (Figures 4A-4F).

**Figure 5:**
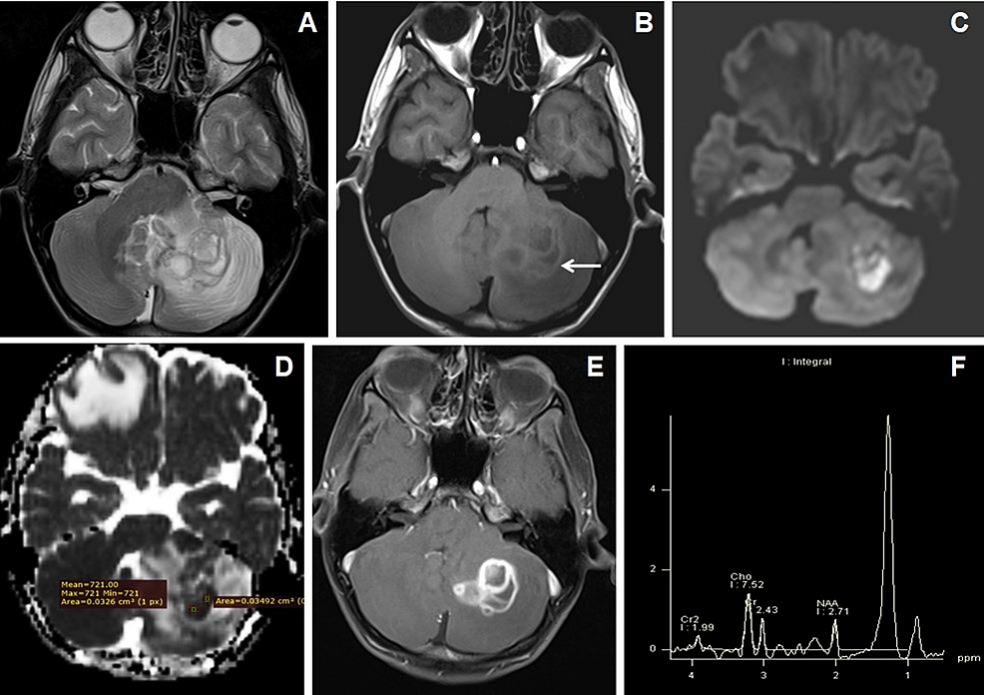
Cranial MRI of a 16-year-old male with headache and seizures due to left cerebellar tubercular abscess in a known case of pulmonary TB. (A and B) Axial T2WI and T1WI images show conglomerated central T2W hyperintense and peripheral hypointense lesions in the left cerebellar hemisphere with mild-to-moderate perifocal edema. T1WI shows peripheral T1WI hyperintensities within the lesions (←arrow). (C and D) Axial DWI and ADC map images show central restricted diffusion with low ADC values. (E) Axial T1W post-contrast image show thick-walled peripherally enhancing conglomerated lesions. (F) MR spectroscopy image at TE-135 ms shows a large lipid peak at 0.9-1.3 ppm and a reduction of NAA peak. NAA: N-acetyl aspartate; DWI: diffusion-weighted imaging; ADC: apparent diffusion coefficient; TB: tuberculosis

**Figure 6:**
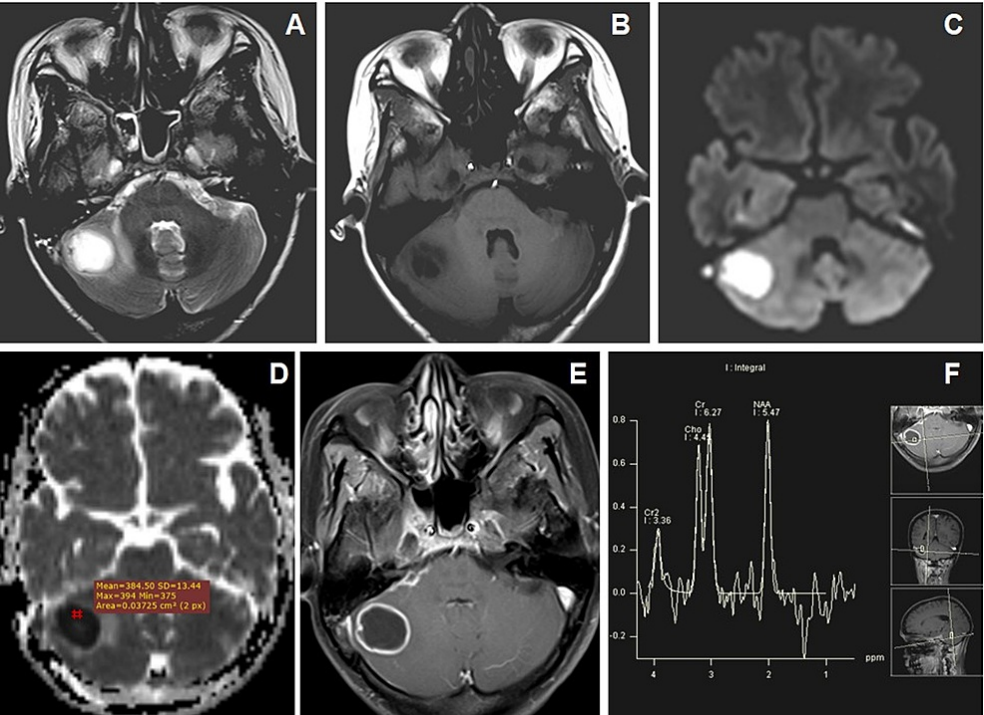
Cranial MRI of a 40-year-old female patient with headache and vomiting with and right-sided ear discharge due to pyogenic abscess in right cerebellum. (A and B) Axial T2WI and T1WI images show central T2W hyperintense with peripheral hypointense wall lesion in the right cerebellum inciting mild perilesional edema. (C and D) Axial DWI and ADC map images show central diffusion-restriction with a low ADC value. (E) Axial T1W post-contrast image shows thick-walled peripheral rim-like enhancement of the lesion with central liquefaction. (F) MR spectroscopy image at TE-135 ms shows an amino acid peak at 0.9 ppm, a lipid-lactate peak at 1.3 ppm, and a doublet succinate peak at 2.4 ppm. DWI: diffusion-weighted imaging; ADC: apparent diffusion coefficient

DWI and MR spectroscopy differentiate tubercular (Figures 5A-5F) and pyogenic abscess (Figures 6A-6F). Pyogenic abscess shows an increased amino acid peak at 0.9 ppm, lipid-lactate peak at 1.3 ppm, and succinate peak at 2.3 ppm (Figure 6) on MR spectroscopy while tubercular abscesses show increased lipid peak at 0.9 ppm with or without lactate peak at 0.9-1.33 ppm (Figure 5).

**Figure 7:**
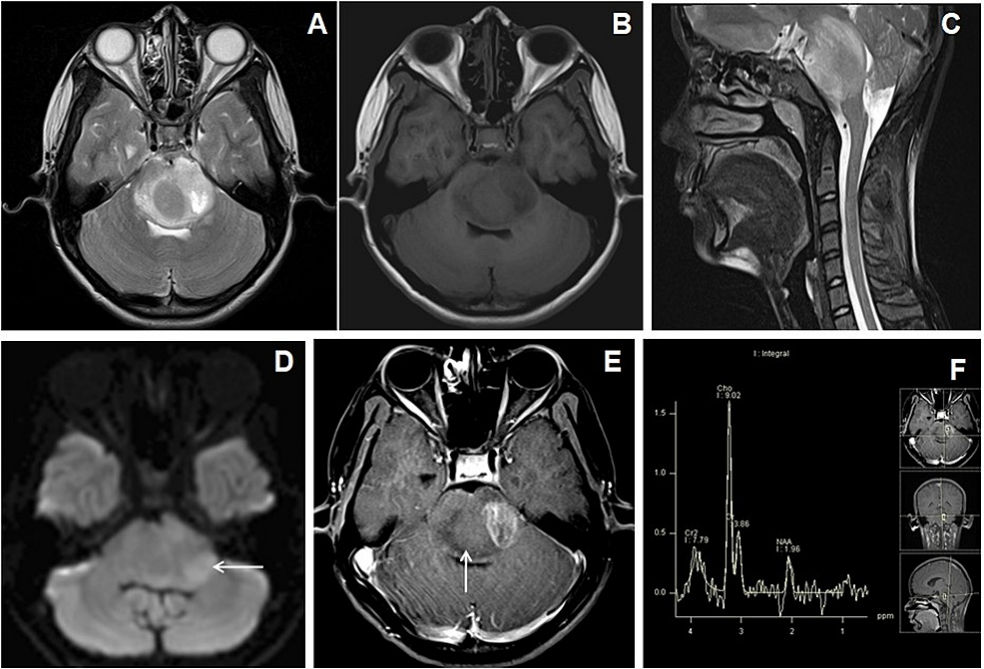
Cranial MRI of a 13-year-old female patient with headache and vomiting due to diffuse brainstem glioma. (A-C) Axial T2WI, T1WI, and sagittal T2WI show central T2W isointense and surrounding T2W hyperintense lesion in the pons, which show central T1W isointensities and peripheral hypointensities. Expansion of the affected brain stem was noted which exerting pressure effect over the fourth ventricle. (D) Axial DWI image shows subtle patchy restriction within the left posterior lateral aspect of the lesion (←arrow). (E) Axial T1W post-contrast image shows patchy heterogeneous enhancement within the left lateral half of the lesion (↑arrow). (F) MR spectroscopy image at TE-135 ms shows increased choline peak and reduction of NAA peak. NAA: N-acetyl aspartate; DWI: diffusion-weighted imaging

Brainstem gliomas showed diffusion restriction with low ADC values with a raised choline, lactate peaks, and Cho/cr ratio on MR spectroscopy (Figures 7A-7F). Two patients each having cerebellar pilocytic astrocytomas and hemangioblastoma showed minimally raised choline peak with a low Cho/cr ratio (less than 1.2) in MR spectroscopy.

**Figure 8:**
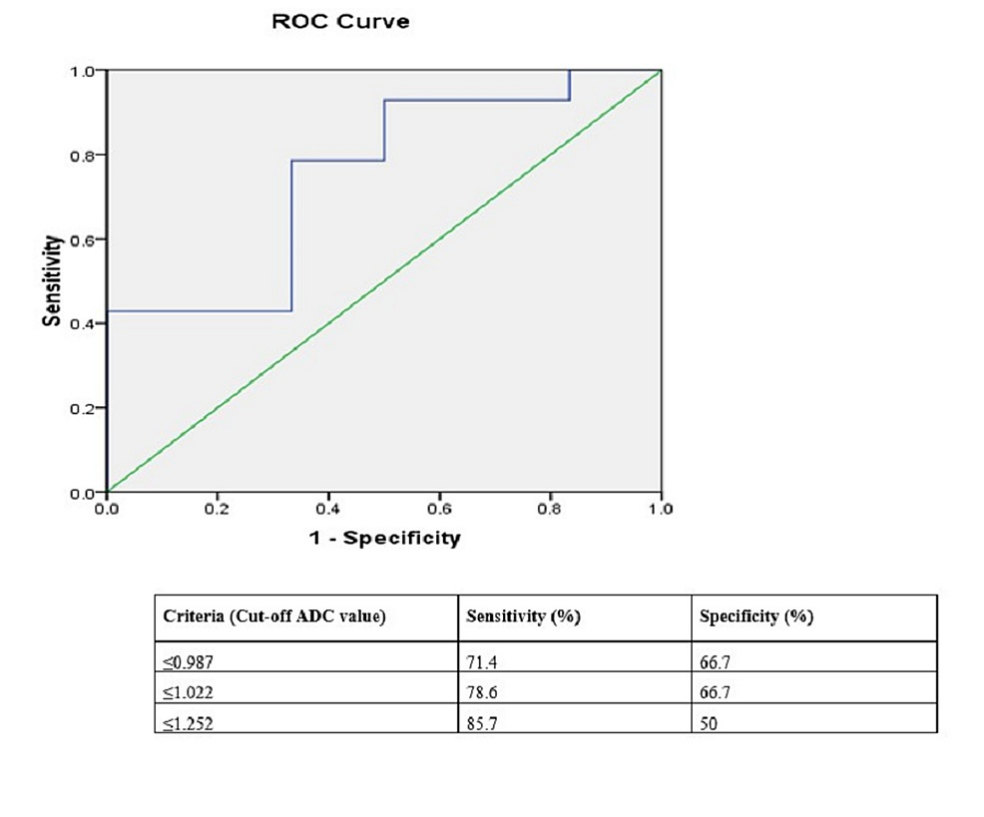
ROC curve analysis for mean ADC values of benign and malignant posterior fossa tumors in 26 patients. ROC: receiver operating characteristic; ADC: apparent diffusion coefficient

Of 26 posterior fossa tumors, 18 (45%) were malignant and eight (20%) were benign tumors. The mean ADC value of benign tumors was 1.279±0.38 × 10^-3^mm^2^/s and malignant tumors was 0.954 ±0.27× 10-^3^mm^2^/s with a statistical significance of p = 0.019. The cut-off mean ADC value was 1.022 × 10^-3^mm^2^/s with a sensitivity of 78.6% and specificity of 66.7% (Figure 8).

**Figure 9:**
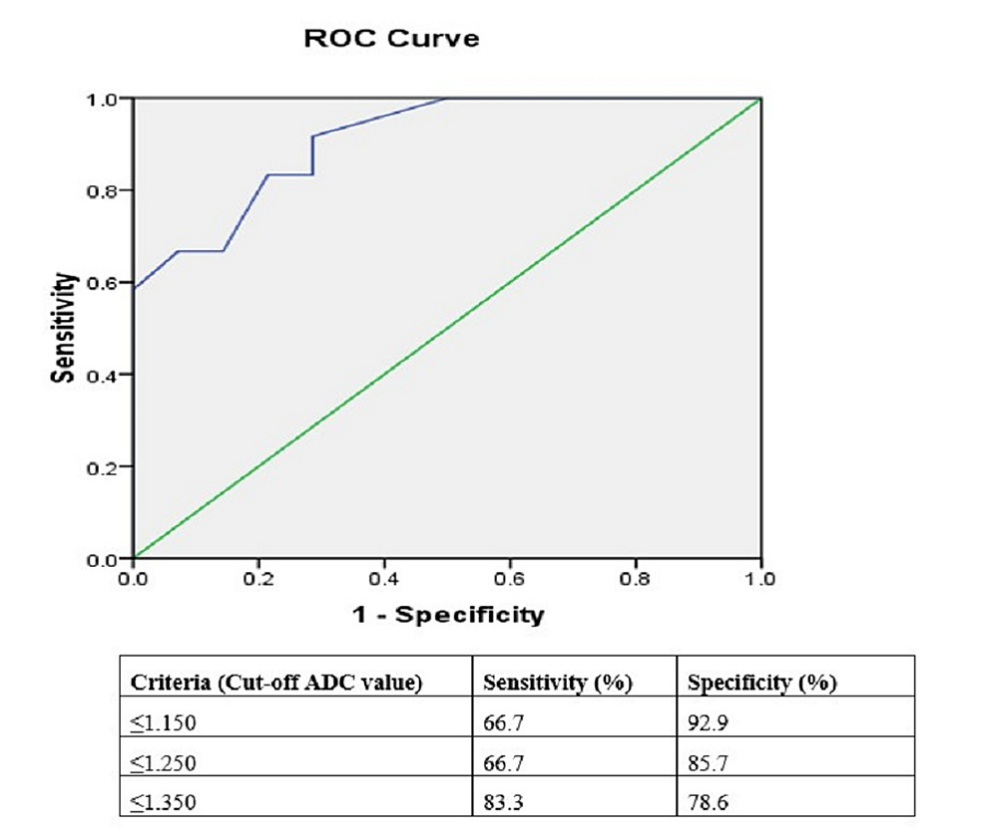
ROC curve analysis for Cho/cr ratio in benign and malignant posterior fossa tumors in 26 patients. ROC: receiver operating characteristic; Cho/cr ratio: choline/creatinine ratio

The mean Cho/cr ratio for benign and malignant posterior fossa tumors were 1.01 ± 0.24 and 1.96 ± 0.42 respectively with a statistically significant difference (p =0.0005). The cut-off Cho/cr ratio was 1.25 with a sensitivity of 66.7%, specificity of 85.7% (Figure 9).

## Discussion

Forty cases of various posterior fossa space-occupying lesions were evaluated in this study. Tumors, infective lesions, cystic lesions, and tumor-like lesions were considered space-occupying lesions of the posterior fossa.

Metastases were the most common intra-axial lesion to affect the posterior fossa in our study followed by brainstem glioma. In our study, metastatic lesions showed reduced NAA level, elevated choline peak, Cho/cr ratio, and prominent lipid-lactate peak which correlated with the study conducted by Ningapa et al. [8] and Meyer et al. [9]. No elevated choline peak was observed in perilesional edema. High-grade glioma shows similar MR spectral findings, however, there were increased choline peak, Cho/NAA, and Cho/cr ratios in perilesional edema and correlated with previous studies by Law et al. [10], Wang et al. [11], Tsougos et al. [12]. The brainstem gliomas and medulloblastoma showed areas of diffusion restriction with low ADC values along with a raise choline peak and Cho/cr ratio in our study sample [13-16]. Pilocytic astrocytoma and hemangioblastoma did not show diffusion restriction [17].

Vestibular schwannomas diagnosed on basis of its classical location at the opening of the internal auditory canal, extension along the course of the eighth cranial nerve and classic "ice-cream on cone appearance" diagnosed on conventional MR images [18].

Diffusion-weighted imaging with ADC values plays an important role in differentiating arachnoid cyst from an epidermoid cyst in with epidermoid cysts owing to their cellular content exhibiting diffuse restriction with lower to variable ADC values [4,19].

In our study, MR spectroscopy showed prominent amino acid and succinate peaks in pyogenic abscesses, prominent lipid peaks in a tubercular abscess. These MR spectroscopy findings are correlated with previous studies of Karatag and Karatag [20], Gupta et al. [21], Kumar et al. [22], and Meena et al. [23].

Kumar et al. found a good correlation of advanced MRI imaging in the evaluation of posterior fossa tumors with histopathological diagnosis [2]. Darweesh et al. and Moller-Hartmann et al. found a more added value of a combination of MR spectroscopy and ADC values over conventional MR sequences in the differentiation and grading of brain tumors [24,25]. In our study, we found similar observations wherein the use of DWI and MRS increased the diagnostic accuracy of MRI in posterior fossa lesions.

Limitation

The major limitations of our study were the heterogeneous nature of the different space-occupying lesions of the posterior fossa we included and the fewer number of non-neoplastic lesions we encountered in our study sample.

## Conclusions

MRI is the imaging modality of choice for better characterization and differentiation of various space-occupying lesions of posterior fossa lesions because of its superior soft-tissue resolution and multiplanar capabilities. Adding DWI and MR spectroscopy over conventional MRI sequences helps us make the diagnosis much closer to the histopathological diagnosis.
